# The role of infiltrating immune cells in dysfunctional adipose tissue

**DOI:** 10.1093/cvr/cvx108

**Published:** 2017-07-11

**Authors:** Tomasz J. Guzik, Dominik S. Skiba, Rhian M. Touyz, David G. Harrison

**Affiliations:** 1 British Heart Foundation Centre for Excellence, Institute of Cardiovascular and Medical Sciences, University of Glasgow, Glasgow, Scotland, UK;; 2 Translational Medicine Laboratory, Department of Internal Medicine, Jagiellonian University, Collegium Medicum, Krakow, Poland;; 3 Department of Clinical Pharmacology, Vanderbilt University, Nashville, TN, USA

**Keywords:** Inflammation, Hypertension, Adipose tissue, Atherosclerosis, Diabetes

## Abstract

Adipose tissue (AT) dysfunction, characterized by loss of its homeostatic functions, is a hallmark of non-communicable diseases. It is characterized by chronic low-grade inflammation and is observed in obesity, metabolic disorders such as insulin resistance and diabetes. While classically it has been identified by increased cytokine or chemokine expression, such as increased MCP-1, RANTES, IL-6, interferon (IFN) gamma or TNFα, mechanistically, immune cell infiltration is a prominent feature of the dysfunctional AT. These immune cells include M1 and M2 macrophages, effector and memory T cells, IL-10 producing FoxP3+ T regulatory cells, natural killer and NKT cells and granulocytes. Immune composition varies, depending on the stage and the type of pathology. Infiltrating immune cells not only produce cytokines but also metalloproteinases, reactive oxygen species, and chemokines that participate in tissue remodelling, cell signalling, and regulation of immunity. The presence of inflammatory cells in AT affects adjacent tissues and organs. In blood vessels, perivascular AT inflammation leads to vascular remodelling, superoxide production, endothelial dysfunction with loss of nitric oxide (NO) bioavailability, contributing to vascular disease, atherosclerosis, and plaque instability. Dysfunctional AT also releases adipokines such as leptin, resistin, and visfatin that promote metabolic dysfunction, alter systemic homeostasis, sympathetic outflow, glucose handling, and insulin sensitivity. Anti-inflammatory and protective adiponectin is reduced. AT may also serve as an important reservoir and possible site of activation in autoimmune-mediated and inflammatory diseases. Thus, reciprocal regulation between immune cell infiltration and AT dysfunction is a promising future therapeutic target.

## Introduction

Physiologically, adipose tissue (AT) stores energy to support metabolic requirements in the times of need. From an evolutionary point of view, this is beneficial, but with increased nutrient intake and reduced energy expenditure in our modern world, AT function becomes altered leading to obesity.^1^ Such alteration is a result of complex interactions of metabolic and immune factors. Understanding of the importance of immunity in metabolic regulation, and the role of metabolism in immune regulation, underlies the rapidly developing biological field of immunometabolism. For example, T cell or M1 macrophage activation is typically associated with a switch from oxidative phosphorylation to anaerobic glycolysis.^2^ This has been reviewed in depth elsewhere,^3^^,^^4^ and, in the present paper, we will focus on the role of interactions of immune cells with dysfunctional AT.

AT can be typically classified as white, brown, or beige based on its metabolic activity, number of mitochondria, and uncoupling protein 1 (UCP-1) content, all of which affect adipocyte size and function. Brown AT plays a key role in thermogenesis, while white AT serves primarily for lipid storage. Brown AT is sparse in adult humans, in contrast to its periaortic location in rodents.^5^ In spite of this, the protective properties of brown fat have been demonstrated in cardiovascular disease.^6^ White AT is widely distributed as visceral (VAT) and subcutaneous AT (SAT).^7^ These compartments differ in their functional importance for metabolic health and in their immunometabolic properties. VAT is metabolically more active than SAT and it harbours significantly more immune cells in both health and pathology.^8^ This is closely linked with increased glucose uptake and fatty acid generation in VAT and greater adrenergic innervation, all of which are important in the regulation of insulin sensitivity.^7^ SAT in turn absorbs circulating free fatty acids and triglycerides.^7^ Numerous studies have shown that the retroperitoneal content of VAT is linked to cardiovascular risk.^9^ This is mediated by chronic low-grade inflammation, characterized by an excessive immune cell infiltration, overproduction of detrimental adipokines and cytokines (TNF-α, IL-6) that can be detected systemically as biomarkers of inflammation.^10^^,^^11^ Mechanistically such low-grade inflammation alters metabolic functions of AT, leading not only to insulin resistance and diabetes but also to cardiovascular pathology.^12^^,^^13^ More recently, attention has been focused on a very specific compartment of VAT, the perivascular AT (pVAT), due to its close proximity to blood vessels and its unique embryonic origin from vascular smooth muscle cell SM22+ precursors.^8^ Dynamic interplay between white and beige/brown adipocytes within pVAT results in unique metabolic and pro-inflammatory properties that make pVAT an important regulator of vascular function and plaque stability.^8^ Human perivascular coronary adipocytes exhibit reduced differentiation, more irregular shape, and smaller size than in the SAT or typical peri-renal VAT. This translates into smaller lipid droplet accumulation and increased synthetic capacity.^14^ pVAT provides a microenvironment for recruitment and activation of immune cells which in concert with adipokines affect vascular tone and other aspects of vascular homeostasis.^15–17^

**Figure 1:**
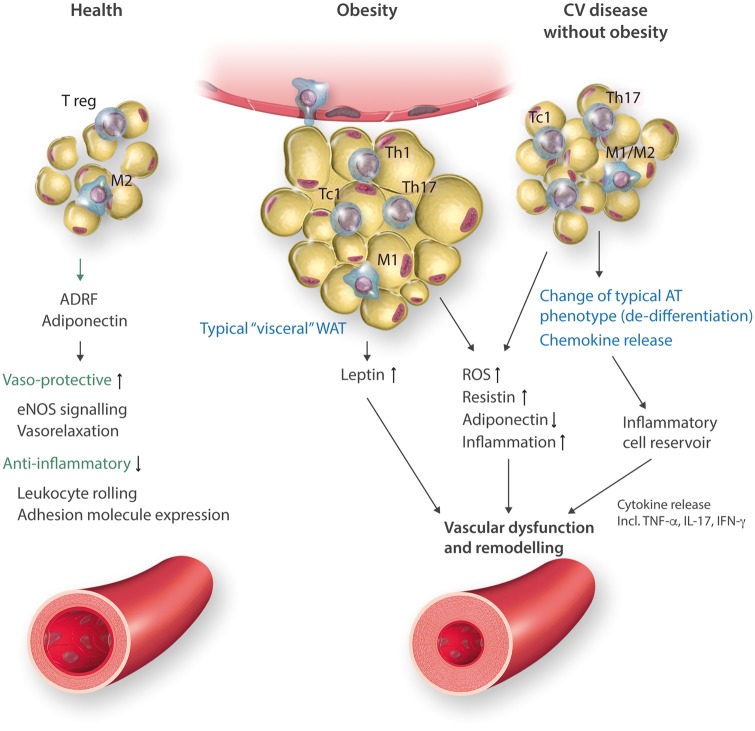
Triple functions of adipose tissue (VAT/pVAT) in health, obesity and in cardiovascular (CV) disease without obesity. AT compartments differ in characteristics of infiltrating immune cells, characteristics of adipocytes and adipokine profile. In health, protective adipokines and cytokines are important in maintaining vascular homeostasis. In obesity, enlarged adipocytes produce leptin and do not release adiponectin and enhance M1 macrophage accumulation in crown-like structures as well as T effector cells. In CVD without obesity macrophages are atypical, adipocytes are synthetic and create microenvironment for development of TLOs and immune cell activation.

In summary, all compartments of AT: SAT, VAT as well as pVAT serve physiological functions in vascular and metabolic homeostasis. When these protective functions are disturbed, *dysfunctional AT* promotes the development of metabolic and vascular disease (*Figure 1*).

## Physiological roles of immune cells in AT

In health, AT contains numerous cell types, including not only adipocytes but also endothelial cells, fibroblasts, pre-adipocytes, stem cells, and regulatory/naive immune cells.^18^ Immune cells including M2 macrophages and T regulatory cells (Treg) release anti-inflammatory cytokines such as interleukin (IL)-10 and transforming growth factor beta (TGF-β), which increase insulin sensitivity and inhibit AT inflammation and dysfunction (*Figure 1*).^19^ In lean conditions, M2 cells are characterized by a lack of CD11c and the presence of CD206 and arginase 1.^20^ M2 and Treg polarization are reciprocally enhanced in physiological conditions by adiponectin released from IAT.^21^ IL-10 modulates insulin signalling through insulin receptor/IRS1-IRS2/PI3-kinase/Akt/FOXO1, in the context of hepatic gluconeogenesis and lipid synthesis. These actions are partially direct and in part indirect, through modulation of TNF, IL-6, IL-1β, and M1 macrophage polarization.^22^ M2 macrophages control adipocyte lipolysis.^23^ Upon cold exposure, M2 macrophages secrete catecholamines, to stimulate adipocyte lipolysis. This is important because, in concert with eosinophils, M2 macrophages can orchestrate generation of beige AT.^24^ As discussed above, in lean, insulin-sensitive AT T cells present are primarily T regulatory cells that secrete IL-10 and transforming growth factor-β (TGFβ) and Th2 cells producing anti-inflammatory cytokines such as IL-4, IL-5, IL-13, and IL-10. These play an important role in homeostasis of AT.^25^ Tregs in normal AT have a unique mRNA expression profile, characterizing their regulatory function, such as CD25, glucocorticoid-induced tumor necrosis factor receptor (GITR), cytotoxic T lymphocyte antigen-4 (CTLA-4), killer cell lectin-like receptor G1 and OX40 in addition to classical FoxP3.^25^ T regs also exhibit chemokine sensitivity as evidenced by high CC chemokine receptor expression.^25^ Other immune cells in lean AT include potentially protective eosinophils and to a lesser extent neutrophils. To date, the role of these cells has been less well defined. Likewise, the role of immune cells present in healthy pVAT in the regulation of vascular function has not yet been clearly defined, apart from potential effects on the release of protective adipokines from adipocytes. Immune cell content in lean subcutaneous AT has also been described but is very low. Dynamic changes of immune cells in the AT underpin their involvement in pathologies associated with AT dysfunction.

## Defining dysfunctional AT

Functional changes within the AT associated with altered paracrine and endocrine properties contribute to the development of cardiovascular disease and cancer.^26^^,^^27^*ATdysfunction* is thus characterized by decreased release of homeostatic protective factors such as adiponectin, nitric oxide, or protective prostaglandins and increased activation of stress-related pathways leading to pathological adipokine release (resistin, visfatin, leptin) and development of low-grade inflammation (*Figure 1*),^28^ which is not only a feature of dysfunctional AT but also promotes metabolic and vascular dysfunction. While this phenomenon is particularly evident in pVAT, it has also been well defined in other VAT depots^26^^,^^29^ in obesity.^8^ Adipocyte–immune cell interactions are therefore bi-directional and depend on nutritional mechanisms, neuro-hormonal pathways, and locally secreted humoural factors.^8^^,^^26^^,^^29^ In pathological conditions, adipocytes produce inflammatory cytokines and extracellular matrix proteins, supporting infiltration and activation of immune cells, therefore, creating an optimal microenvironment for inflammation.^5^ At the same time, activated immune cells secrete cytokines that influence adipocyte function, and differentiation and adipokine secretion. Links between adipokines and immune cell infiltration in the AT have been discussed elsewhere and are summarized in *Table 1*. The characteristics of AT inflammatory responses differ between classical inflammatory disease such as Crohn’s disease and cancer or cardiovascular disease. Common feature is, however, that dysfunctional, inflamed AT provides a microenvironment permissive for the development of pathology. These effects can be localized, for example linking pVAT to adjacent vessel dysfunction in hypertension or atherosclerosis^38^^,^^39^ or systemic, such as the effects of VAT dysfunction on the development of diabetes, cancer, autoimmune diseases, or signalling within the CNS.

**Table 1:** Summary of the effects of adipokines on immune responses. Expertly reviewed and discussed elsewhere.^30,31–37^

Adipokine	Immune cell recruitment	Immune cell activation	Summary
Leptin	↑ CCL3, CCL4 and CCL5 from Mf Directly stimulates Mo/Mf chemotaxis through canonical pathways	Similar to IL-2 ↑ IL-6/TNF in Mo/Mf ↑ T cell activation (CD69+/CD25+) and proliferation ↑ Th1 (IL-2/IFNg) ↑ Th17 and ↓Treg ↓ Th2 (IL-4) ↓ NK cell cytotoxicity	Pro-inflammatory
Adiponectin	↓ Eo chemotaxis ↓ ICAM-1 in EC ↓ CXC chemokine ligands (e.g. IP-10) and T cell recruitment	↓ IL-17 production from γ/δ T cells ↑ IL-8 in synovial fibroblasts ↓ Antitumour DC immunity Mf activation resembling M1 (but with M2 elements; ↑mannose receptor) ↑ CD4 T cell activation	Anti – inflammatory via AdipoR1 receptor; In some conditions pro-inflammatory^34^
Resistin	↑MIP-1β, GRO-α and CCL1 in Mf ↑CX3CL1 and CX3CR1 direct chemotaxis of human CD4+	Expressed in Mf and T cells Induced by IL-1/IL-6/TNF ↑ IL-6, IL-27, IL-23 and IL-5 in Mf (↑) Th17 and Th1	Pro-inflammatory
Visfatin *(PBEF-1)*	↑ICAM-1; VCAM-1 on EC and VSMC	↑ B-cell maturation ↑ Leukocyte activation ↑ TNF/IL-6/IL-1b ↑ NFkB	Pro-inflammatory
Chemerin * (RARRES2* or *TIG2)*	Direct chemotaxis through CMKLR1; chemR23 especially on DCs; NK; Mf	↓TNF/IL-6/ ↑ NFkB ↑ Adiponectin ↑ TGFβ	Pro-inflammatory and anti-inflammatory
RBP4	?	Activates APCs in AT inflammation and T cell activation Inhibited by TNF	Pro-inflammatory?

Eo, eosinophil; Mf, macrophage, Mo, monocyte, NK, natural killler cells; EC, endothelial cells; Th, T helper; CD, cluster of differentiation; IL, interleukin; TNF, tumour necrosis factor alpha; CCL, CC chemokine ligand; CXCL1, fraktalkine; PBEF-1, pre-B-cell colony-enhancing factor – visfatin; *TIG2, tazarotene-induced gene 2*; RARRES2, retinoic acid receptor responder protein 2; CMKLR1, chemokine like receptor 1.

## Immune cells in AT dysfunction

**Figure 2:**
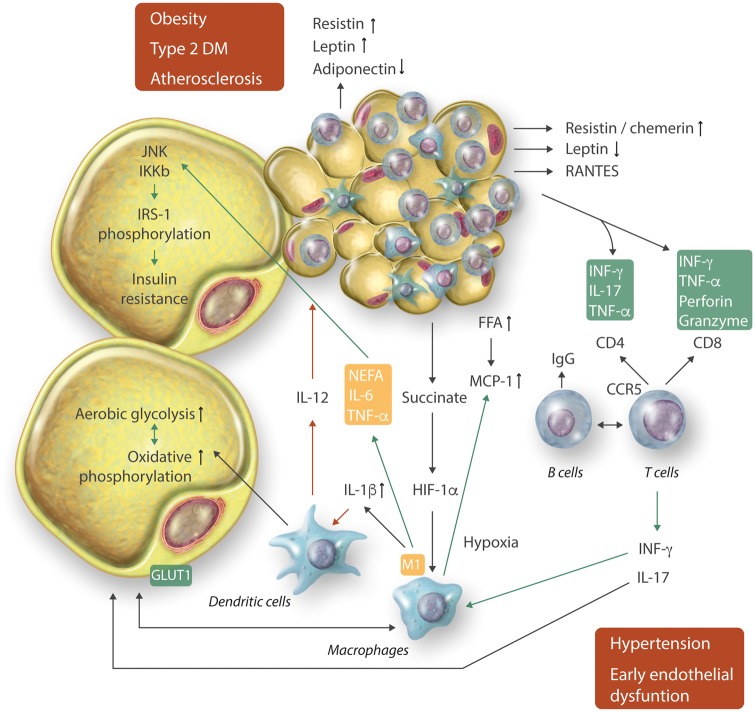
Interactions between adipocytes and immune cells at different stages of metabolic and cardiovascular disease. Interactions involve important immunometabolic regulation.

Immune cells that infiltrate dysfunctional AT are the key drivers of AT inflammation (*Figure 2* and *Table 2*). The cellular players of such responses differ depending on the anatomical location as well as on the type and the stage of pathology.^77^^,^^78^

**Table 2:** Key cell types infiltrating adipose tissue in health and disease – selected metabolic and cardiovascular (CV) effects. See Table 1 legend for abbreviations

Cell type		Preferential localisation	Metabolic effects	Role in CV pathology
Macrophages Antigen Presenting Cells (DCs)		VAT>pVAT^38^ VAT>SAT^40^	Insulin resistance (M1) Higher AT ROS production^41^ Increased lactate production^41^ Regulate differentiation of adipocytes via GM-CSF signalling^42^ ATMs can inhibit adipogenesis^43^	Polarising M1 phenotype in atherosclerosis and hypertension Role in hypoxia Promote vascular Th17 response^44^ M2 Mf in vascular fibrosis^45^
T cells	CD8+	VAT>SAT^40^	Insulin resistance^46^ Cause steatohepatitis^47^ Regulate glucose tolerance via perforin^48^	initiate inflammatory cascades^46^ role in macrophages differentiation, activation and migration^46^ impair vascular function^39^
	Th1	VAT>SAT^49^^,^^50^	• Promote insulin resistance^48^	• impair vascular function^39^Promote atherosclerosis^51^^,^^52^
	Th17	Epi.AT>Ing.AT^53^ VAT>SAT	Associated with cholesterol level^54^ Promote insulin resistance^53^ Promote diabetes and autoimmune responses enhance obesity^55^; Suppress adipocyte differentiation^53^	Hypoxia^54^ Increased inflammation^54^ IL17 increases ICAM1^54^ Contributes in foam cells formation^54^ Increased atherosclerosis^56^^,^^57^
	Th2	VAT>SAT^49^^,^^50^	Improve glucose tolerance via IL-13/STAT3 and M2 induction Enhance beiging^24^	• Improve vascular function; Increase or decrease atherosclerosis^58–60^
	γ/δ T cells	VAT>SAT^53^	Promote insulin resistance^61^	• Induce vascular dysfunction and hypertension^62^role in atherosclerosis unclear^63^
	Tregs	VAT>SAT^40^^,^^64^	Insulin sensitivity^65^ Improve glucose tolerance^65^	Decrease vascular inflammation^65^ Prevent atherosclerosis^52^^,^^66^^,^^67^
B cells		pVAT>VAT^7^VAT>SAT^40^	Glucose intolerance mediated by IgG^68^ Higher fasting insulin level^68^	Higher production of IgG^68^ Activate vascular CD8+ and Th1 cells^68^ promote atherosclerosis^52^
NK cells		VAT>SAT^69^Epi.AT>Ing.AT^70^	• Insulin resistance^69^	Differentiation to M1 macrophages^69^ INF-γ production^69^ Impair vascular function^71^
NKT cells		Epi.AT>Ing.AT^70^	Insulin resistance^72^ Hepatic steatosis^47^^,^^72^	• Contribute to vascular production of IFN-γ, IL-4, and TNF-α^72^
Eosinophils		VAT>SAT^73^	Insulin sensitivity^73^ Reduce body weight^24^ Increase beiging^24^	IL-4 and IL-13 release perivascularly (Th2) Polarization of M2 macrophages^73^—possibly profibrotic In pVAT—anti contractile; improve vascular function^74^
Neutrophils		VAT>SAT^75^	Insulin resistance^76^ Decreased adiposity^76^	Increase of vascular M1 macrophages^76^ Decrease of vascular M2 macrophages^76^

Macrophages were the first immune cells identified in AT.^79^ They are also the most abundant cell type in typical visceral and subcutaneous AT, representing more than 50% of all leukocytes. Their content in SAT is several folds lower than in typical VAT in both health and disease, suggesting their metabolic role. Resident AT macrophages (ATMs) play immune and scavenger functions. They present antigens to lymphocytes, phagocytose foreign organisms, release antimicrobial peptides, and attract other immune cells to areas of inflammation.^10^^,^^80^ In lean animals and humans, ATMs characterized by the surface markers F4/80 or CD68 constitute less than 5% of all AT cells. A dramatic increase (up to 40% of all AT cells) is observed in metabolic stress.^10^^,^^81^ Such an increase is also associated with qualitative changes of ATMs. In lean AT, M2-like producing IL-10 macrophages are dispersed, while in dysfunctional AT, M1 macrophages predominate and form crown-like aggregates, surrounding necrotic adipocytes/lipid droplets.^13^^,^^20^^,^^82^ In pathological conditions, these classically activated, M1 polarized, CD11c+ macrophages increases,^83^ produce pro-inflammatory TNF-α and IL-6 and IL1β.^13^^,^^84^ Such simple dichotomous division of ATMs into protective M2 and damaging M1 cells appears to be an oversimplification, especially when it concerns human pathology. Several studies point to the role of M2 cells in dysfunctional AT and insulin resistance^82^ or vascular remodelling and fibrosis^45^ indicating the need for further phenotypic characterization of ATM that may include Ly6C, CD34, CCR2, and CX3CR1.^85^ Macrophages also promote further propagation of AT inflammation through numerous humoural and cellular mechanisms including release of metalloproteinases such as ADAMTS13 and others.^77^^,^^86–89^ Discussion continues what proportion of these cells is chemotactically recruited and what proportion is proliferating from resident ATMs.^90^^,^^91^

Other types of innate immune cells in VAT and pVAT include neutrophils, representing about 2% of visceral stromal, non-adipocyte, cell fraction. In contrast to resident macrophages and dendritic cells (DCs), their presence may be transient,^75^ but they may still contribute to insulin resistance^76^ (*Table 2*). Especially, in lean conditions, AT harbours eosinophils and mast cells, cells that are typically involved in allergic reactions. Eosinophils secrete IL-4 and IL-13 and contribute to the anti-inflammatory, insulin-sensitive AT phenotype that supports the expansion of M2 ATMs.^73^ Their content in pathology is decreased. Mast cells in turn increase in dysfunctional AT and have been linked to atherosclerosis and metabolic dysfunction^92^ by promoting monocyte recruitment.^93–95^

**Figure 3:**
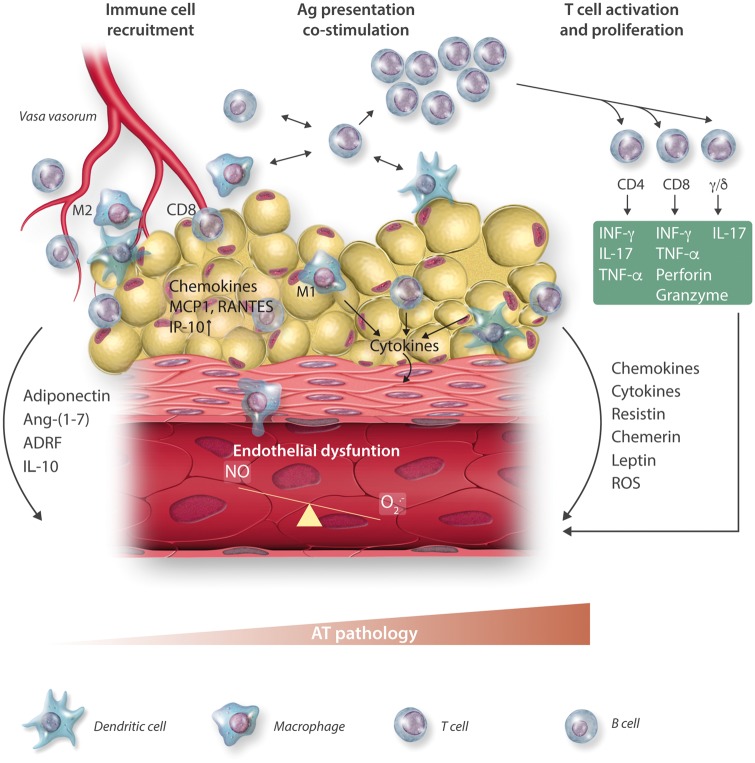
Perivascular AT inflammation as a mechanism of endothelial dysfunction.

While the role of macrophages in AT dysfunction is predominantly linked to their innate functions, these cells also serve as antigen-presenting cells leading to the activation of the adaptive immune system in AT. This is particularly evident in pVAT, where tertiary lymphoid structures have been identified.^96^^,^^97^ Dendritic cells, which are the most efficient antigen presenting cells, have also been identified both in typical VAT^98^ and in pVAT.^8^^,^^38^^,^^39^ Thus, dysfunctional AT, creates a microenvironment permissive for T and B lymphocyte activation,^98^ and lymphocytes constitute the second most abundant immune cell population in VAT.^99^ In some diseases, their content in the AT exceeds the number of macrophages^38^^,^^39^ allowing for the propagation of inflammation.^100^^,^^101^ T cells that expand in pathology and promote development of insulin resistance, atherosclerosis, and hypertension include predominantly IFN-γ-producing Th1 (CD4+) and Tc1 (CD8+) cells, producing IFNγ and TNF, and IL-17 producing Th17 cells (*Figures 1* and *3*). These cells initiate an inflammatory cascade that may precede ATM infiltration.^46^ Another subset of T cells, key to AT dysfunction, include invariant natural killer T (iNKT) cells (*Table *2). These lymphocytes express a semi-invariant TCR and proteins typical of NK cells but recognize lipid and glycolipids presented in the context of CD1d MHC-like molecule.^102^ They can produce both Th2- and Th1-type cytokines.^103^ In healthy human omentum, up to 10% of T cells are iNKT cells and their number is reduced in patients with obesity and cancer.^104^ Their exact role is not fully recognized but link to immune activation by lipids makes them a critical candidates for important immuno-metabolic cells.^105^ Recently, gamma-delta (γ/δ TcR) T cells have been demonstrated to represent substantial proportion of T cells in the AT and their number increases in metabolic and vascular pathologies.^61–63^^, ^^106^ Importantly, these cells are an important source of strongly pro-inflammatory Il-17 and may further regulate immune responses. T cell presence and activation in dysfunctional AT is also closely linked to inflammasome activation.^107^ Nlrp3 in regulates IL-18 and IFN-γ in the AT and promotes effector T cell accumulation in AT.^107^ Finally, there is a small number of B cells in the VAT of lean animals, where they provide immunity against infections, including bacteria from peritoneal space.^108^ B-cell content increases in dysfunctional AT, where they promote activation of other immune cells and may affect metabolic status (*Table 2*).

The mechanisms of immune cell recruitment and the metabolic and functional consequences of their presence in AT vary in different pathological conditions which are briefly summarized below.

## Immune cells in the AT and metabolic diseases

**Obesity**

Increased adipocyte size triggers a stress response and release of chemoattractant proteins, such as MCP-1, M-CSF-1, or RANTES,^109^ leading to monocyte recruitment and macrophage accumulation.^10^^,^^11^^,^^110^ As discussed above, Adipokines also induce chemokine expression and have key chemotactic properties themselves (*Table 1*).^109^ There is a correlation between the accumulation of AT macrophages and adipocyte size.^10^ Local lipid fluxes are also regulators of ATM recruitment.^111^ High levels of free fatty acids (FFA) elevate chemokine secretion from adipocytes inducing macrophage chemotaxis to VAT. FFAs activate TLR4 signalling in adipose cells. In TLR4 knockout mice, AT inflammation is prevented, and these animals are protected against obesity-induced insulin resistance.^112^ Finally, hypoxia and oxidative stress in the VAT is characteristic for obesity and can promote chronic inflammation through metabolic and classical chemokine-dependent mechanisms.^113^^,^^114^ Apart from chemotaxis, increased macrophage proliferation^115^^,^^116^ and differentiation from preadipocytes can enhance the content of macrophages.^117^ Obesity and insulin resistance are characterized by the predominance of M1 macrophages in the VAT.^13^^,^^84^ Mechanisms of M1 macrophage polarization in obesity are not entirely clear. Non-esterified fatty acids (NEFA) are produced in AT and increased systemically in obese subjects. NEFA induce the expression of IL-6, while reducing IL-10 (*Figure 2*).^118^ In contrast, PPARγ skews macrophages toward an alternative M2 phenotype by regulating fatty acid storage and, in doing so, reduces obesity and improves insulin resistance (*Figure 2*).^119^

While the metabolic state plays a role in macrophage recruitment and polarization, ATMs in turn have important effects on AT metabolism (*Figure 2*).^3^ Depletion of macrophages in AT increases the expression of adipose triglyceride lipase (ATGL) and genes regulated by FFAs. Blockade of monocyte recruitment to VAT genetically or pharmacologically, through CCR2 antagonism protects from diet-induced obesity, improves insulin sensitivity, and lowers AT genes expression related to inflammation and AT dysfunction.^81^^,^^84^^,^^120^ Similarly, selective depletion of M1 macrophages decreases pro-inflammatory genes expression and reduction in crown-like structures in obese AT, and consequently improves insulin sensitivity.^121^ Weight loss decreases macrophage content leading to improved insulin sensitivity.^111^ Both fasting and bariatric surgery^111^^,^^122^ decrease MCP-1, CSF-3, and genes related to hypoxia (HIF1-α) in AT and consequently reduce the number of ATM cells.^122^

While macrophages are quantitatively the most abundant immune cells in obesity, T cells also play a critical regulatory role.^99^ They increase significantly in the AT in obesity and tend to localize around enlarged adipocytes.^123^ T cells can interact with ATMs regulating inflammatory responses and metabolic dysfunction.^124^ Of importance are the cytotoxic CD8+ T cells that secrete TNF-α, IL-2, IFN-γ, and chemokine RANTES and CD4+ Th1 cells that secrete TNF-α, IL-12, and INF-γ. These cytokines directly affect adipocyte function and promote M1 macrophage polarization.^125^ T cell recruitment in obesity is partially mediated by the RANTES–CCR5 axis.^99^^,^^123^ T cell infiltration of AT may precede macrophage-dependent inflammation as it is present after 4–5 weeks of high-fat feeding while macrophage influx was observed after 10 weeks.^126^ AT T cells infiltration is strongly associated with early reduction of insulin sensitivity and impaired glucose tolerance.^126^ In line with this, CD8^−^^/^^−^ mice are protected from M1 macrophage AT infiltration and subsequent AT dysfunction in obesity.^46^ Indeed, T cell cytokines are essential for macrophage polarization in the setting of classical inflammation.^127^ A specific subset of pro-inflammatory T cells (CD153 + PD-1 + CD44hiCD4+) are remarkably increased in the VAT of HFD-fed mice. These osteopontin-producing CD4+ T cells show functional and genetic features of senescent T cells.^128^^,^^129^ T cells in obese AT are regulated by NLRP3 inflammasome, which senses obesity-associated danger signals and contributes to obesity-induced inflammation and insulin resistance.^107^^,^^130^ These mechanisms also link macrophage activation to T cell role in obesity.

Other immune cells are also increased in AT in obesity. B cell AT infiltration is associated with increased IgG production in the AT. Concentrations of pro-inflammatory IgG2c in serum and VAT are elevated in obese mice. Most importantly, B cells from obese mice transferred into B cell-deficient lean mice induce insulin resistance.^68^ Apart from antibody-mediated mechanisms, B cells from obese mice secrete pro-inflammatory cytokines (IL-6 and INF-γ) and can directly regulate T cells and macrophages.^131^

Eosinophils also play an important role in the immune regulation of obesity. Mice lacking eosinophils exhibit weight gain, insulin resistance, and increased proinflammatory M1 macrophages in the AT.^73^ At the same time, mice with eosinophilia (overexpressing IL-5) demonstrate decreased adiposity and improved insulin sensitivity when fed a high-fat diet.^73^ IL-5 can be produced by AT itself but importantly by innate lymphoid type 2 cells (ILC2s). Deletion of ILC2s causes significant reductions in VAT eosinophils and alternatively activated macrophages M2. Interleukin 33, which promotes activation and recruitment of the ILC2s, leads to ILC2-dependent increases in VAT eosinophils and M2 macrophages.^132^ Finally, the role of iNKT cells in obesity is not clear. While they are activated by lipid, iNKT cell number is decreased in obesity^104^ and their depletion increases fat deposition, enhances the presence of M1 macrophages in VAT, and increases insulin resistance and glucose intolerance. Adoptive transfer of iNKT cells into obese mice causes weight loss, improvement of glucose tolerance, and insulin sensitivity.^133^

A link between vascular oxidative stress and obesity in the context of insulin resistance was recently reported in mice with vascular smooth muscle-targeted deletion of p22phox subunit of NADPH oxidase.^134^ High-fat feeding did not induce weight gain or leptin resistance in these mice which was associated with strongly reduced T-cell infiltration of pVAT. This is important as indicates causal immunometabolic linking vascular dysfunction to obesity suggesting that vascular inflammation may be primary in the development of obesity and insulin resistance.^134^^,^^135^ Such wide-spread participation of various immune cells in metabolic regulation demonstrates the complexity of the immune system and AT inflammation in obesity.

**Diabetes and insulin resistance**

Immune cell infiltration into AT provides an important link among obesity, insulin resistance, and diabetes. The number of macrophages infiltrating AT in obese patients with insulin resistance is higher than in patients with insulin-sensitive obesity, independent of the fat mass.^11^ Insulin levels affect AT inflammation during high-fat diet.^11^ Progressive macrophage infiltration in VAT preceded increase of insulin in serum, suggesting that AT inflammation is a cause rather than the consequence of insulin resistance.^11^ Increasing evidence supports the role of adaptive immunity in insulin resistance and diabetes, through inducing pro-inflammatory cytokines in metabolic organs, such as the AT, liver, muscle, and pancreas.^136^ CCR5 knockout mice are protected from insulin resistance induced by high-fat diet and this effect is mediated by reduced effector T cell accumulation with subsequent reduction of ATMs and M2 polarization of persisting macrophages.^137^ Clinical studies confirmed that Th1 cells are up-regulated in the AT and peripheral blood from patients with prediabetes or T2DM.^138^ Moreover, high fat diet and insulin resistance are associated with accumulation of Th1, Th17, and effector CD8+ lymphocytes in the AT, while anti-inflammatory Th2 and Treg cells are decreased.^125^ Combined anti-CD3 and glucosylceramide treatment induces IL-10 and TGF-β, reducing VAT inflammation in obese mice, and improving fasting glucose levels.^101^

Immune cell activation, involving the co-stimulatory molecule CD40 and its ligand CD40L, is particularly important in linking AT inflammation to diabetes.^139^ CD40–CD40L interactions promote pancreatic, AT, and vascular inflammation (*Figure 3*),^140^^,^^141^ increasing the expression of pro-inflammatory cytokines and chemokines (e.g. TNF-α, IL-6, MCP-1), leukotriene B4 at the same time enhancing lipid droplet accumulation and adipogenesis.^142–144^ These effects are mediated by reduced expression of insulin receptor substrate (IRS-1) and glucose transporter type-4 (GLUT-4).^140^^,^^143^ CD40L expressed on T cells may induce AT inflammation and impair insulin sensitivity (*Figure 2*).^140^

## AT immune cells in vascular disease—hypertension and atherosclerosis

**Hypertension**

Hypertension represents an important example of immuno-metabolic vascular disease.^145–147^ It is associated with obesity and BMI is one of the strongest predictors of increased blood pressure. Many hypertensive subjects are not obese, but present features of metabolic dysregulation. In hypertension with or without obesity, pVAT inflammation is a prominent feature, and is involved in the pathogenesis of vascular dysfunction.^39^ This leads to the loss of protective properties of pVAT and promotes loss of endothelium-dependent vasodilatation and enhanced vasoconstriction.^8^ These functional changes are linked with morphological alterations, as pVAT becomes synthetic, pro-inflammatory, often de-differentiated, and highly metabolically active (*Figure 3*). This profile is characterized by changes in adipokines (increased resistin and visfatin and decreased adiponectin and leptin) and increased production of chemokines such as RANTES or IP-10 (CXCL10) that are key for recruitment of activated monocytes/macrophages and CD8+ T cells. Apart from AT-specific factors activating immune system in the pVAT, central nervous system is also involved,^148^ which is important in the context of high perivascular sympathetic innervation and its role in hypertension.^149^

In health, the immune cell infiltrate in the pVAT constitutes only about 2% of the stromal vascular fraction (SVF) cells.^38^^,^^39^ In vascular pathologies, such as Ang II-induced hypertension, leukocytes in pVAT increase to 7–10% of SVF cells, and, in atherosclerosis, their content reaches up to 10–20%. Hypertension is linked with a significant increase of T cell and antigen presenting cell pVAT infiltration, which mediates endothelial dysfunction^150^ and provides a link between hypertension and subsequent atherosclerosis. Dysfunctional endothelium promotes inflammation through a number of NFkB dependent, Notch/Jagged1-regulated integrin, and adhesion molecule expression.^151^^,^^152^ Both CD4+ and CD8+ T cell subpopulations are increased in the pVAT in hypertension and express higher levels of proinflammatory cytokines (TNF-α, INF-γ) and CCR5.^39^^,^^153^^,^^154^ T cell activation and vascular and renal recruitment is essential for the development of AngII-induced hypertension.^153^ This is partially mediated by RANTES, similar to obesity and insulin resistance, through which Th1, Tc1, and gamma-delta (γ/δ) T cells, lymphocytes are recruited to the vascular wall.^39^ Th17 cells, essential for blood pressure increase, are in turn recruited in a RANTES-independent CCR6, -dependent manner.^62^ Th17 cells not only participate in blood pressure increase^155^ but also contribute to vascular stiffening observed in hypertension.^156^ In contrast, adoptive transfer of suppressive, Tregs prevent AngII-induced hypertension and vascular inflammation and improves vascular function.^157^^,^^158^ B cells in pVAT are almost equal in percentage of SVF cells to T cells and their number is increased during hypertension.^39^ They may act as antigen-presenting cells, modulating T cell responses, and produce IgG2b and IgG3. Depletion of B cells protects from hypertension.^159^ Finally, macrophage infiltration is also significantly increased in hypertensive pVAT.^39^ Elevated blood pressure is correlated with pVAT expression of macrophage chemokine receptors CCR2 and its ligands CCL2, CCL7, CCL8, and CCL12. Moreover, the CCR2 antagonist INCB3344,7–9 reduces CCR2 expression and reverses macrophage accumulation in pVAT of mice with hypertension.^160^ Macrophages in pVAT in healthy conditions appear to be predominantly unpolarised or skewed towards M2.^38^^,^^39^ However, when blood pressure is elevated, the level of both M1 and M2 subpopulations is increased.^39^ Macrophage infiltration to the pVAT during hypertension is regulated by T cell-dependent mechanisms^39^ as lymphocyte adaptor protein (LNK) deficiency, leading to hyperactivated T cells increased number of macrophages in the aorta and pVAT.^161^

Classical antigen-presenting cells such as DCs are regulators of adaptive immune response may play an important role in initiation of inflammation by interactions with T cells. They occur in small numbers in pVAT in the healthy state and their number increases during hypertension.^39^ Elevated oxidative stress leads to endogenous peptide modification by isoketal (isolevuglandin) adduct formation. This occurs in AT, vessels, and kidneys and promotes antigen presentation by dendritic cells precipitating the role of the T cells in hypertension and further development of pVAT inflammation.^162^ Blocking the co-stimulation molecules between T cells and dendritic cells prevents pVAT inflammation and decreases blood pressure.^163^ Moreover, DCs secrete cytokines such as IL-1β, IL-6, IL-23 which promote polarization of T lymphocytes to Th17 cells, which plays particular role in hypertension development.^155^ Thus, hypertension and associated vascular dysfunction result from complex interactions between several cell types involved in inflammatory responses in hypertension. All types of cells discussed above coexist together in pVAT and they can interact with each other initiating inflammation and causing development of vascular dysfunction and disease.^8^

The effector mechanisms linking infiltrating immune cells to AT dysfunction in hypertension are related to the release of effector cytokines such as IL-17A, IFNγ, TNF-α, and IL-6.^20^^,^^164^ These cytokines also impair endothelium-dependent relaxation as demonstrated in *ex vivo* studies,^39^ as well as *in vivo* using INF-γ knockout mice.^71^^,^^165^ IL-6 is also necessary for Th17 cell differentiation.^166^ IL-17, a key pro-hypertensive cytokine, is a potent activator of the endothelial cells promoting the expression of adhesion molecules.^167^ IL-17A activates RhoA/Rho-kinase and increases inhibitory eNOS Thr495 phosphorylation in endothelial cells leading to decreased NO production.^168^ Inflammatory cytokines modulate smooth muscle cell constriction, proliferation, and migration.^169^ They also affect adipokines release from AT. For example, TNFα, IL-6, and IL-17A can all inhibit expression and release of adiponectin.^170–172^ One of the key adipokines, leptin, has a structure similar to IL-6, IL-12, IL-15 and can affect leukocyte activation and chemotaxis, release of oxygen radicals, VSMC proliferation, and expression of adhesion molecules on endothelial and vascular smooth muscle cells.^173^ IL-17A and TNF increase leptin and resistin production in AT which upregulate the expression of VCAM1 and ICAM and/or induction of CCL2 as well as endothelin-1 from endothelial cells^174^ and can induce vascular dysfunction and oxidative stress.^8^^,^^135^ All these mechanisms, besides promoting pVAT dysfunction, provide a link between hypertension and atherosclerosis, in part independently of blood pressure.

**Atherosclerosis**

PVAT is dysfunctional at all stages of atherogenesis. Increased levels of chemerin, visfatin, leptin, and vaspin are correlated with atherosclerosis development.^175^ At early stages of atherosclerosis macrophages, T cells and dendritic cells are recruited into perivascular adventita and AT surrounding vasculature.^38^ This precedes development of endothelial dysfunction^176^ and oxidative stress^110^^,^^177^ and can be modified by interventions targeting numerous metabolic functions such as Ang(1-7).^38^^,^^178^ Such perivascular inflammation of AT continues to be observed at later stages of the disease, with further increase of macrophage and B cell content.^179^^,^^180^ In a pivotal early study, Galkina et al. observed high leukocytes number in aorta with pVAT in old ApoE^−^^/^^−^ mice in advanced atherosclerosis.^179^^,^^180^ Perivascular inflammation, in particular T cell dependent, correlates with lesion size and is clearly age dependent,^180^^,^^181^ and T cell depletion prevents atherosclerosis.^182^ Leukocyte infiltration to pVAT in atherosclerosis is mediated by similar mechanisms to those observed in hypertension. IL-8, RANTES, and MCP-1 are all increased in the pVAT from arteries with atherosclerotic plaques.^183^ We have recently described a key role of increase in M1 macrophage polarization in early atherosclerosis in the pVAT and measures to reduce pVAT M1 macrophage differentiation prevent plaque formation.^38^ Pro-inflammatory IL-17A-producing T cells are present in the adventitia and blockade of IL-17A leads to reduction of macrophage accumulation and atherosclerosis.^184^ At early stages, leukocytes are scattered throughout the PVAT,^179^^,^^180^ however, with age they seem to organize to form perivascular arterial tertiary lymphoid organs (ATLO),^96^^,^^97^ which can serve also suppressive functions or become dysfunctional. Molecular mechanisms of pVAT inflammation in atherosclerosis indicate several key targets linking immune responses to metabolic dysfunction. Signal transducer and activator transcription 4 (STAT4) is expressed in adipocytes and immune cells and can participate in PVAT inflammation. STAT4 deficiency reduces development of atherosclerosis and PVAT inflammation in ApoE^−^^/^^−^ mouse and in insulin resistant obese Zucker rats.^185^ Interestingly, the number of CD8+ T cells is increased in pVAT of Apoe-/-mice indicating that in metabolic disease, hypertension, and atherosclerosis CD8 cells play a particularly important regulatory role. Recently, an important regulatory function has been attributed to myeloid-derived suppressor cells that can affect AT inflammation.^186^ Finally, the role of B cells has recently been clarified in atherosclerosis. B cells may serve as an important source of antibodies which promote plaque inflammation and development but can also contribute to antigen presentation and are important source of humoural factors such as TNF.^187^ The complexity of immunity of atherosclerosis is reviewed elshewhere.^182^^,^^188^

## AT immune cells in immune and inflammatory disorders

Autoimmune and inflammatory diseases are typically associated with metabolic dysregulation.^189^ This is particularly evident in psoriasis, ankylosing spondylitis and rheumatoid arthritis and is linked with development of metabolic syndrome. Psoriasis is associated with significant perivascular, global arterial, and SAT inflammation.^190^ Similarly, AT in rheumatoid arthritis is highly infiltrated with macrophages which form crown-like structures. These macrophages are activated and express mixed characteristics with high levels of TNF, IL-1beta, but also IL-10.^191^ These macrophages secrete chemokines (CCL2 and RANTES) as well as IL-6, IL-8, MMP-3.^191^ These factors further promote macrophage infiltration and can mediate T cell recruitment and activation. T regulatory cells resident in AT may serve an important role in maintaining self-tolerance, and their impairment may promote development of autoimmunity.^192^ This mechanism may link epidemiological suggestions of links between obesity and autoimmune diseases.^192^ A key unanswered question is whether adipose tissue in autoimmune disease can create a microenviroment for T cell activation and participate in the pathogenesis of autoimmune disease, or if it is a mere manifestation of systemic inflammation.

## Ectopic fat depots and chronic inflammation

Ectopic AT is the visceral fat surrounding intraabdominal organs and located in the liver, heart, pancreas, and muscles. Its presence is linked to low-grade inflammation and cardio-metabolic complications commonly experienced in type 2 diabetes.^9^ In particular, non-alcoholic fatty liver disease constitutes an important risk determinant for cardiometabolic risk. Myocardial triglyceride, epicardial, and pericardial fat depots accumulate with increasing amount of liver fat and VAT.^193^ Thus, the association of LV diastolic function with hepatic ectopic fat may be an indicator of systemic inflammation. Ectopic fat accumulation in the liver is linked to the infiltration of the γ/δ+ T cells, granulocytes, and CD11b+ cells in mice. It appears that IL-6 regulates recruitment of these cells and IL-17 production in the liver that promotes ectopic fat.^194^ This is in part regulated by decreased microRNAs (miR) such as miR26a, providing a link to cardiac injury.^195^ Similar regulatory properties have been attributed to other miRs expressed in the AT and cardiovascular system.^49^^,^^196–199^ The inflammatory nature of epicardial AT has been known for years,^200^ and is supported by numerous molecular mechanisms.^196^ Only recently, however, have we started appreciating the heterogeneity of epicardial AT which is particularly linked to its pro-inflammatory properties.^30^^,^^201^ It may also underlie a link between subclinical atherosclerosis and epicardial fat thickness and hepatic steatosis.^202^ Thus, ectopic fat accumulation in and around the heart, kidneys, muscles, and liver is a marker of increased cardiovascular risk likely linked to chronic inflammation. At the same time, through the release of adipokines and chemokines, it attracts pro-inflammatory cells like IL-17 producing γ/δ+ T cells, which contribute to the pathology.

## Translational evidence

While most of data regarding immune cell infiltration of AT originate from animal models, the role of immune cells has been clearly demonstrated in humans. Similar to animal models, macrophages constitute about 4% of the total AT stromal visceral fraction and it increases up to 15% in obesity.^203^ There are, however, some key differences in the characteristics of immune cells infiltrating human AT. In contrast to animal studies, an ‘M2-type’ macrophage with remodelling capacity (e.g. through TGF-β and IL-10 release), but also able to secrete proinflammatory cytokines, has been identified in obese AT in humans.^204^ These mixed-type macrophages have CD11c^+^CD206^+^ characteristics but are pro-inflammatory and linked with insulin resistance in human obesity.^82^ T cell infiltration in human AT is much less characterized.^99^ AT T cells correlate with BMI, their recruitment is dependent on RANTES chemokine and functionally affects adipocyte and pre-adipocyte differentiation and function.^99^ Detailed characteristics, activation mechanisms, and effector functions of effector T cells present in human AT are still poorly defined. Adipokines have been shown to regulate human immune cell activation, for example inhibit IL-17 production from T cells and CD8+ effector cell accumulation (summarized in *Table 2*).

Interestingly, several studies have recently shown that vascular dysfunction, may regulate AT dysfunction, with immune cell infiltration as a key intermediate step. For example, p22phox overexpression in VSMCs leads to increased diet induced obesity that is mediated by AT T cell infiltration.^134^ The same has been shown in humans where oxidative stress derivated such as 5-HNE regulate adiponectin release from AT.^50^^,^^205^^,^^206^ Significant weight loss, in obese individuals, demonstrates clear links to reduced immune cell infiltration in the AT with concomitant improvement of insulin sensitivity and vascular function.^122^ Several clinical studies using immune targeted therapies in patients with type 2 diabetes confirmed experimental suggestions of the causal role of inflammation in insulin resistance and hyperglycaemia. Indeed, in patients with type 2 diabetes treated with IL-1 receptor blocker (Anakinra),^207^ IL-1β antagonist (gevokizumab,^208^ canakinumab,^209^ LY2189102^210^), TNF antagonist (CDP571,^211^ Ro 45-2081,^212^ etanercept^213^) or IKKβ-NF-κB inhibitor^214^ all have been shown to improve metabolic profile providing an important translational evidence.

## Conclusions

Over the years, it has become apparent that vascular and metabolic dysfunction occur in a wide range of vascular pathologies and are closely regulated by coincident immune dysregulation. Immune cells infiltrating AT both sense and can induce metabolic disturbances, contributing to a vicious circle of AT dysfunction. Immune infiltration of AT is critical in T2D, obesity or insulin resistance it is also a primary feature of hypertension or atherosclerosis, making immuno-metabolic interventions a valuable therapeutic approach in a wide range of cardiovascular pathologies. While in animal models of metabolic disease, we have now identified the key immune cell subpopulations and their immunometabolic profiles, relatively little is known about human AT infiltration. One challenge is to identify specific immune cell populations within human AT that could be targeted and differences in their characteristics depending on anatomical location. Finally, we need to understand dynamic changes of the role of immune cells at different time points of metabolic and vascular pathology.

While specific therapeutic interventions limiting AT inflammation may be designed based on this,^215^^,^^216^ we already know that commonly used agents, including methotrexate, anti-TNF therapies and leflunomide limit macrophage infiltration in AT.^217^ Similarly, several vasoactive therapies such as ACE-inhibitors or angiotensin II receptor blockers have potential to limit inflammation in pVAT. While these approaches lead to systemic immunosuppression, more specific small molecule immune targeted therapies might prove helpful to improve the metabolic profile of AT and prevent AT dysfunction.
